# Temporal and spectral analyses of EEG microstate reveals neural effects of transcranial photobiomodulation on the resting brain

**DOI:** 10.3389/fnins.2023.1247290

**Published:** 2023-10-17

**Authors:** Nghi Cong Dung Truong, Xinlong Wang, Hanli Liu

**Affiliations:** Department of Bioengineering, University of Texas at Arlington, Arlington, TX, United States

**Keywords:** transcranial photobiomodulation (tPBM), electroencephalogram (EEG), EEG microstate, empirical mode decomposition, Hilbert-Huang transform

## Abstract

**Introduction:**

The quantification of electroencephalography (EEG) microstates is an effective method for analyzing synchronous neural firing and assessing the temporal dynamics of the resting state of the human brain. Transcranial photobiomodulation (tPBM) is a safe and effective modality to improve human cognition. However, it is unclear how prefrontal tPBM neuromodulates EEG microstates both temporally and spectrally.

**Methods:**

64-channel EEG was recorded from 45 healthy subjects in both 8-min active and sham tPBM sessions, using a 1064-nm laser applied to the right forehead of the subjects. After EEG data preprocessing, time-domain EEG microstate analysis was performed to obtain four microstate classes for both tPBM and sham sessions throughout the pre-, during-, and post-stimulation periods, followed by extraction of the respective microstate parameters. Moreover, frequency-domain analysis was performed by combining multivariate empirical mode decomposition with the Hilbert-Huang transform.

**Results:**

Statistical analyses revealed that tPBM resulted in (1) a significant increase in the occurrence of microstates A and D and a significant decrease in the contribution of microstate C, (2) a substantial increase in the transition probabilities between microstates A and D, and (3) a substantial increase in the alpha power of microstate D.

**Discussion:**

These findings confirm the neurophysiological effects of tPBM on EEG microstates of the resting brain, particularly in class D, which represents brain activation across the frontal and parietal regions. This study helps to better understand tPBM-induced dynamic alterations in EEG microstates that may be linked to the tPBM mechanism of action for the enhancement of human cognition.

## 1. Introduction

Over the past decade, photobiomodulation (PBM) has attracted substantial interest as a practical method for treating a variety of pain and/or infections using low-dose red to near-infrared (630–1,100 nm) light. Examples of PBM applications include pain alleviation (Fulop et al., 2010) and wound healing (Mester et al., 1971; Conlan et al., 1996; Yasukawa et al., 2007; Peplow et al., 2010). Transcranial PBM (tPBM), which refers to PBM administered to the cerebral cortex, has also been proven to boost human cognition (Eells et al., 2004; Barrett and Gonzalez-Lima, 2013; Holmes et al., 2019; Truong et al., 2022) including attentional performance (Jahan et al., 2019) and to be a treatment for traumatic brain injury (Choi et al., 2012; Figueiro Longo et al., 2020), Alzheimer’s disease (Grillo et al., 2013; Nizamutdinov et al., 2021), and Parkinson’s disease (Quirk et al., 2012; Liebert et al., 2021). A recent, comprehensive study by Zhao et al. reported significant enhancements in visual working memory capacity in healthy humans through four experiments using two separate laser wavelengths (850 and 1,064 nm) and two stimulation sites (left and right forehead; Zhao et al., 2022).

The mechanism underlying tPBM has been proposed to involve cytochrome c oxidase (CCO), a crucial component in mitochondria responsible for energy generation. The photochemical reactions of CCO initiate a cascade of biochemical events, which were believed to enhance cellular energy production, promote neuronal metabolism, and modulate neurovascular coupling (Rojas et al., 2012; Lee et al., 2017). To better understand the neurophysiological effects of tPBM on the human brain, different imaging modalities have been simultaneously employed, including electroencephalography (EEG; Wang et al., 2019; Ghaderi et al., 2021; Shahdadian et al., 2022), functional magnetic resonance imaging (fMRI; Vargas et al., 2017; Dmochowski et al., 2020), broadband near-infrared spectroscopy (bbNIRS; Tian et al., 2016; Wang et al., 2017; Pruitt et al., 2020; Wang et al., 2022a), and functional near-infrared spectroscopy (fNIRS; Holmes et al., 2019; Truong et al., 2022).

EEG is a widely used and effective measurement tool for noninvasive monitoring of human neurophysiological activity in neuroscience research and clinical applications. In a subset of EEG research, EEG microstate analysis is an established method for investigating brain dynamics in the resting state (Lehmann et al., 1987). EEG microstates are defined as global patterns of scalp potential topographies that dynamically alter over time in an ordered manner. Specifically, spontaneous EEG activity during the resting state can be described by a limited number of EEG topographical maps that remain stable for a short period (60–120 ms). These specific global scalp maps were obtained by spatial clustering of whole scalp topographies without considering the polarity inversion (Pascual-Marqui et al., 1995; Koenig et al., 1999; Koenig and Brandeis, 2016). Briefly, scalp topographies with high spatial correlation independent of polarity were first clustered into one representative topographical map, forming a class of microstates (Michel and Koenig, 2018). A dynamic train or alteration of the microstates is then found by fitting the template maps (or classes) back to the temporal data.

Recent publications have demonstrated that tPBM enables significant alterations in EEG spectral power across the human cortex (Zomorrodi et al., 2019; Wang et al., 2021) and in functional connectivity across several resting-state brain networks (Zomorrodi et al., 2019; Ghaderi et al., 2021; Shahdadian et al., 2022). However, previous EEG-based studies have not investigated the influence of tPBM on the temporal dynamics of the human brain. To the best of our knowledge, only a short conference abstract by Zomorrodi et al. (2021) has reported the effects of tPBM on the temporal dynamics of the human brain. Therefore, it remains unclear how tPBM dynamically modulates the human brain. Accordingly, this study addressed two key questions: can tPBM modulate EEG microstates and their topographical spectral parameters? If so, which temporal and spectral parameters of microstates would tPBM modulate significantly? We hypothesized that tPBM significantly affects the parameters of certain microstate classes.

This study shared the 64-channel EEG data reported earlier (Wang et al., 2021; Shahdadian et al., 2022; Wang et al., 2022b), comprising 45 healthy subjects undergoing both active and sham 8-min tPBM using a 1,064-nm laser applied to the right forehead. The novelty of this study differs from our prior work on several key analysis methodologies and findings. For the first time, EEG microstate analysis (Michel and Koenig, 2018) has been applied to investigate brain dynamics under tPBM. Different temporal microstate parameters were extracted and compared to assess the effects of tPBM on temporal dynamics in the human brain. In addition, EEG microstate spectral analysis was performed using multivariate empirical mode decomposition (MEMD; Lang et al., 2018) and the Hilbert-Huang transform (HHT; Huang et al., 1998; Huang and Wu, 2008). This newly developed frequency-domain analysis enabled us to quantify the alteration in the EEG power of microstate classes over different experimental periods for both tPBM and sham sessions. By the end of this study, our statistical results revealed significant changes in the occurrence, contribution, and transition probabilities of different microstate classes as well as alterations in frequency-band-specific microstate power, which affirmed our hypothesis.

## 2. Materials and methods

### 2.1. Participants

We recruited 49 healthy human subjects (29 males, 19 females, 26 *±* 8.8 years of age) from the University of Texas at Arlington local community to participate in this study. Participants had to be satisfied the following criteria: (1) no psychiatric disorder or neurological condition, (2) no severe brain injury, (3) no history of violence or imprisonment, (4) no current intake of any psychotropic medicine, (5) no smoking or excessive alcohol consumption, (6) had not been diagnosed with diabetes, as required by the laser’s manufacturer (Cell Gen Therapeutics LLC, Dallas, Texas). Due to observed fatigue or drowsiness during EEG measurements, four subjects were excluded from the dataset, leaving 45 remaining participants in the subsequent data analysis. The experimental protocol was approved by the Institutional Review Board of the University of Texas at Arlington. Prior to all measures, each participant’s informed consent was obtained.

### 2.2. Experimental procedures

**Figure 1:**
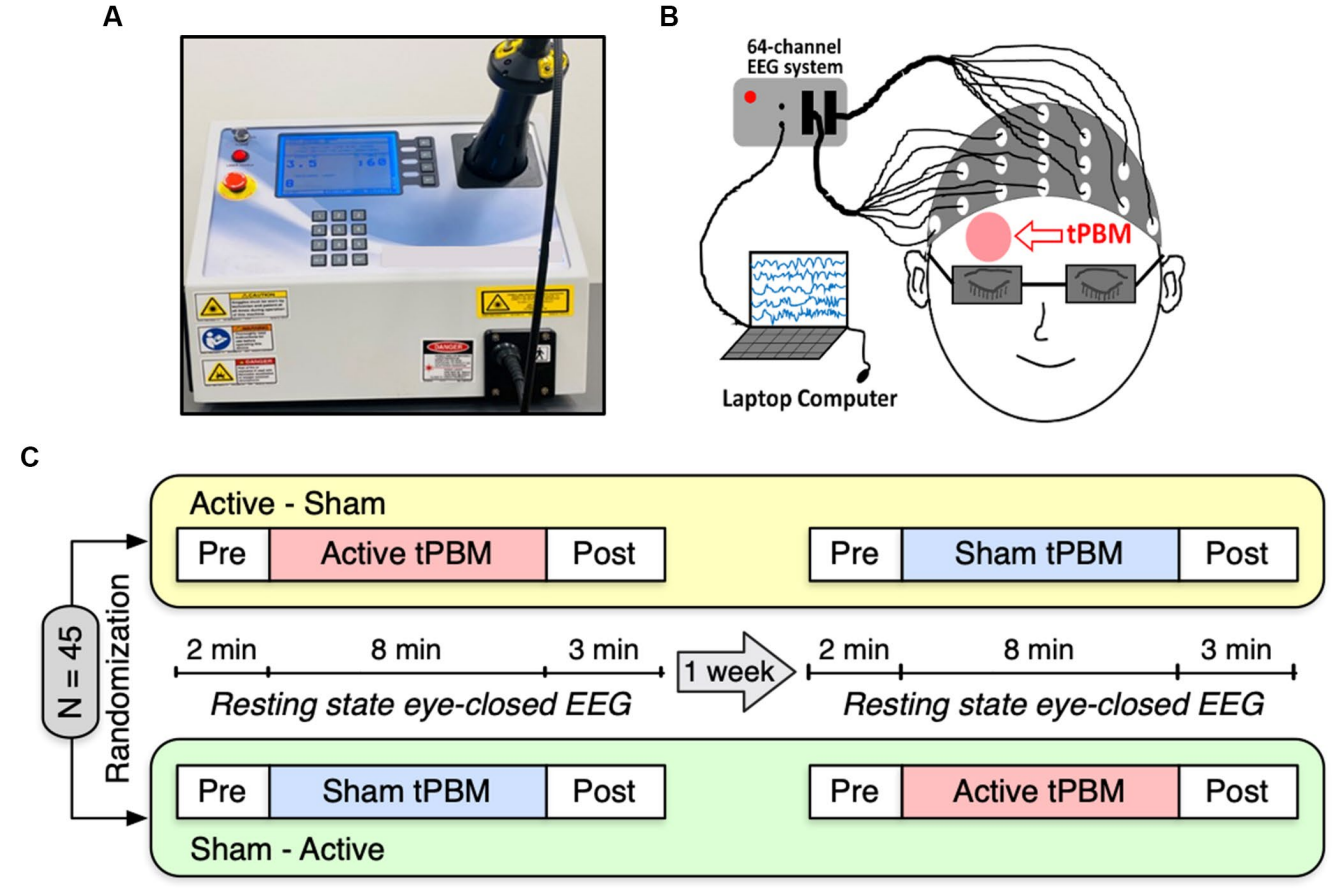
**(A)** A photograph of our 1,064-nm laser used for the study. **(B)** A cartoon showing the EEG setup and the tPBM site on the participant’s right forehead. **(C)** Schematic diagram of the experimental protocol. A total of 45 subjects were randomly divided into two groups: active-sham or sham-active stimulation. Each experiment included EEG recordings of a 2-min baseline, an 8-min active or sham tPBM, and a 3-min recovery period. A minimum 1-week waiting period between two visits was required to avoid potential effects from active tPBM.

We employed a 1,064-nm continuous-wave (CW) laser with FDA clearance (Model CG-5000 Laser, Cell Gen Therapeutics LLC, Dallas, Texas) for our noninvasive tPBM experiment (Figure 1A). The laser had a 13.6 cm^2^ irradiation area, and its power was set at 3.4 W. Using this laser’s output, a total energy dose of 1,632 J was delivered throughout an 8-min tPBM session (3.4 W *×* 60 s/min *×* 8 min = 1,632 J), resulting in a laser power density of 0.25 W/cm^2^. The light was delivered over the right frontopolar region close to the Fp2 site without physical contact (Figure 1B). For the sham condition, the laser device was also on to ensure subjects heard the device operation sound and were unaware of being in the sham condition. However, the laser strength was reduced to 0.1 W for sham stimulation, and a black cap was placed in front of the laser aperture to obstruct the light further. Participants would not be aware of the cap as it was placed after they closed their eyes. Throughout the experiment, participants were required to wear a pair of laser protection goggles.

Figure 1C depicts the experimental protocol. Each subject was assigned a random order for the two study sessions: active tPBM and sham tPBM. In order to prevent any carry-over effects, two visits had to be separated by at least 1 week. Subjects were instructed to sit comfortably with their eyes closed during EEG acquisition. The resting state EEG data were recorded for 2 min of pre-stimulation, 8 min of active/sham stimulation, and 3 min of post-stimulation. We used a Biosemi (64-channel) 10–10 EEG equipment to acquire the EEG data (Figure 1B). The electrical gel was applied to each electrode prior to each EEG measurement in order to boost conductivity and the signal-to-noise ratio of the collected data.

### 2.3. EEG data analysis

#### 2.3.1. EEG data preprocessing

We employed the EEGLAB toolbox (Delorme and Makeig, 2004) to preprocess 64-channel EEG data. Since either 256 or 512 Hz was used to acquire the EEG data, the 512 Hz data were first down-sampled to 256 Hz to ensure consistency. The EEG signals underwent bandpass filtering between 1 and 70 Hz using the EEGLAB “filtfilt” function. Additionally, a notch filter at 60 Hz was employed to remove line noise. Re-referencing was performed by subtracting the average voltage signals across all 64 electrodes from each of the EEG time series. The Independent Component Analysis (ICA) technique (Campos Viola et al., 2009) was applied to eliminate artifacts caused by eye blinks, eye movements, or jaw clenches. ICA components were manually inspected, and the noisy components corresponding to the noise and artifacts were excluded. Subsequently, the artifact-free EEG time series were split into four temporal segments to better characterize the EEG microstates in response to tPBM/sham stimulation: (1) a 2-min pre-stimulation (Pre) period, (2) the first 4-min temporal segment during active/sham tPBM (Stim1), (3) the last 4-min segment of active/sham tPBM (Stim2), and (4) a 3-min post-stimulation (Post) period.

#### 2.3.2. EEG microstate analysis in the time domain

**Figure 2:**
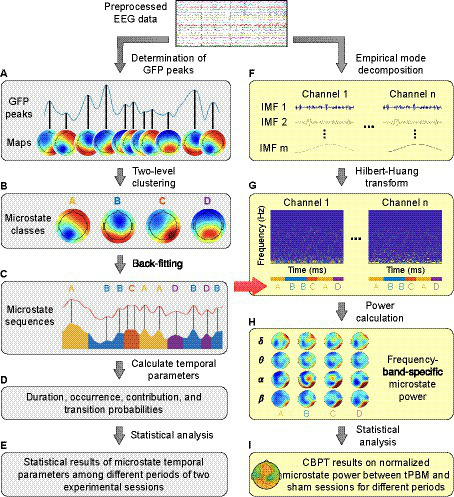
A flowchart for EEG microstate analysis. Panels **(A–D)** show steps of the time-domain EEG microstate analysis; Panels **(F–H)** show steps of the frequency-domain EEG microstate analysis. δ: delta band (0.5–4 Hz); θ: theta band (4–8 Hz); α: alpha band (8–13 Hz); β: beta band (13–30 Hz). Panels **(E,I)** represent the process for statistical analysis in the time and frequency domain, respectively.

The EEG microstate analysis in the time domain was performed following the procedure presented in Koenig et al. (1999) and Michel and Koenig (2018). We employed a Matlab-based microstate toolbox (Koenig, 2017) compatible with EEGLAB (Delorme and Makeig, 2004) to compute EEG microstates. The main steps of the time-domain EEG microstate analysis are depicted by the gray-shaded left column in Figure 2 (i.e., Figures 2A–D) with 4 steps.

The principle of microstate analysis consists of finding a set of the most dominant topographical maps representing different crucial brain states and then fitting these maps back to the EEG data. The global field power (GFP) was calculated for each sample time as follows:


(1) $$\mathrm{GFP}\left(t\right)=\sqrt{\frac{\sum_{i=1}^{N}v_{i}{\left(t\right)}^{2}}{N}}$$

where *N* is the number of EEG electrodes (N = 64 in this study), and *v_i_*(*t*) is the voltage of electrode *i* at time *t*. The time-resolved GFP(*t*) reflects the global power alteration of the EEG signal at time *t*; the GFP peaks correspond to the moments of high global neuronal synchronization (Skrandies, 2007). It is known that the scalp topographies around the peaks remain quasi-stable (Lehmann and Skrandies, 1980; Skrandies, 1989; Koenig et al., 2002; Koenig and Brandeis, 2016; Michel and Koenig, 2018).

In step 1 (Figure 2A), we determined the scalp topographical maps at the GFP peaks for each participant within each experimental temporal period for both tPBM and sham sessions separately.

In step 2 (Figure 2B), we performed two-level clustering to identify global microstates. The first level of clustering was performed to identify the individual-level EEG microstates for each experimental segment (Pre, Stim1, Stim2, and Post), separately for tPBM and sham sessions. All topographical maps acquired per subject per each temporal segment were clustered into 4 maps using the k-means clustering algorithm (Koenig et al., 2002; Murray et al., 2008). These 4 clustered maps present dominant microstate classes, which have been commonly used and reported in numerous EEG microstates studies. Since the individual microstate classes obtained by the k-means clustering had no particular order and thus were potentially mismatched between participants (Koenig et al., 2002; Koenig, 2017), the second level of clustering was performed on EEG microstates of all subjects for each experimental period (Koenig et al., 1999), separately for the tPBM or sham session. The outcome of this clustering was the group-level microstate classes for each experimental period (Pre, Stim1, Stim2, and Post), separately for the tPBM or sham session. Finally, a permutation-based clustering step was employed to identify the “global” microstate classes based on the two groups of 4 microstate classes from the tPBM and sham sessions, serving as representative microstates for all experimental periods of both tPBM and sham sessions.

In step 3 (Figure 2C), the global microstate classes were fitted back to each subject’s temporal EEG data to assign a label of one microstate class to every EEG data instant. The assigned microstate class was chosen as the one that had the highest spatial correlation with the scalp topography of the corresponding EEG data instant (Brodbeck et al., 2012; Michel and Koenig, 2018). The spatial correlation was computed using Pearson’s correlation coefficient (Brandeis et al., 1992) defined as follows:


(2) $$C=\frac{\sum_{i=1}^{N}\left(u_{i}\cdot v_{i}\right)}{\sqrt{\sum_{i=1}^{N}u_{i}^{2}}\cdot \sqrt{\sum_{i=1}^{N}v_{i}^{2}}}$$

where *N* is the number of electrodes, *u_i_* and *v_i_* are the voltage of electrode *i* of the two topographical maps. At the end of Step 3, the labeled microstate time series for all subjects were obtained in both tPBM and sham sessions (Figure 2C).

In Step 4 (Figure 2D), the resulting microstate time series were used to compute four temporal microstate parameters as follows:

- Duration: the average duration that the microstate class is continuously presented (in ms). The microstate duration reflects the average time that the brain sustains synchronized activities.
- Occurrence: the number of occurrences of a microstate class divided by the total duration (in s) of the analyzed EEG data. The occurrence parameter reveals how frequently a microstate class occurs over time (Khanna et al., 2014; Michel and Koenig, 2018).
- Contribution: the proportion of the total occurrence duration of one microstate over the whole analysis time. The contribution parameter indicates the time coverage of each microstate class relative to other classes (Lehmann et al., 2005).
- Transition probability: proportion of the number of transitions from one microstate class to another over the number of all transitions occurring during the analysis period (Lehmann et al., 2005; Khanna et al., 2015).

#### 2.3.3. EEG microstate analysis in the frequency domain

In parallel, we performed EEG microstate analysis in the frequency domain, following the framework proposed in Li et al. (2021). Because conventional time-frequency spectral analysis that employs Fourier or wavelet transform usually fails to analyze EEG signals with a short temporal length [60–120 ms for the case of EEG microstates (Pascual-Marqui et al., 1995; Mandic et al., 2013)], many studies have employed the Hilbert transform in microstate analysis because of its feasibility in analyzing short-length signals (Mandic et al., 2013; Milz et al., 2017; Comsa et al., 2019). Thus, following the methodology proposed in Shi et al. (2020) and Li et al. (2021), we performed a spectral analysis of EEG microstates (Shi et al., 2020) by employing a multivariate empirical mode decomposition (MEMD) algorithm incorporated with the Hilbert-Huang transform (HHT; Huang et al., 1998; Huang and Wu, 2008). The procedure of EEG microstate analysis in the frequency domain is depicted by the yellow-shaded right column in Figure 2 (i.e., Figures 2F–H) in 3 steps, as briefly described below. Detailed mathematical expressions are provided in Supplementary material.

First, MEMD was performed to decompose N-channel EEG signals into a set of intrinsic mode functions (IMFs) that represent different oscillatory levels embedded in the original signals. MEMD is an extended method of EMD, the latter of which is a data decomposition method for non-linear and non-stationary signals (Huang et al., 1998). EMD enables any complicated dataset to be expressed in a finite number of IMFs. MEMD was developed by taking signal projections along different directions in *N*-dimensional spaces, a generalization of EMD (Rehman and Mandic, 2010). This step is illustrated in Figure 2F.

Next, HHT was performed on each of IMFs to estimate the time-frequency Hilbert spectra of all EEG channels and to facilitate the sharp identification of imbedded structures. In addition, the EEG microstate sequences obtained by time-domain analysis (Figure 2C) were imported to generate the segmented Hilbert spectra for each EEG microstate (Figure 2G). Given that the Hilbert spectrum is written as *H^n^*(ω, *t*) for the EEG data from the *n*th channel in the frequency *ω* at time *t*, the power for microstate *m* at the *n*th channel in the frequency band (*<fb>*) would be equal to:


(3) $$P_{\langle fb\rangle }^{mn}=\frac{1}{\Delta \omega }\frac{1}{L_{m}}\iint_{\Delta \omega L_{m}}H^{n}{\left(\omega ,t\right)}^{2}dtd\omega$$

where *L_m_* is the total temporal length of the microstate *m*, and ∆ω is the range of the frequency band *<fb>*. Specifically, *<fb >* covers delta band (δ: 0.5–4 Hz), theta band (θ: 4–8 Hz), alpha band (α: 8–13 Hz), and beta band (β: 13–30 Hz). This step is depictured and marked in Figure 2H.

Finally, the percentage changes of the power for microstate *m* during tPBM/sham and post-tPBM/sham periods with respect to the pre-stimulation (baseline) power were defined as follows:


(4) $$\Delta P_{\langle seg\rangle ,\langle fb\rangle }^{mn}=\frac{P_{\langle seg\rangle ,\langle fb\rangle }^{mn}-P_{pre,\langle fb\rangle }^{mn}}{P_{pre,\langle fb\rangle }^{mn}}\times 100\%$$

where *<seg>* covers three temporal segments (Stim1, Stim2, and Post). These percentage changes were calculated for both tPBM and sham sessions.

#### 2.3.4. Statistical analysis

For the time-domain microstate analysis (Figure 2E), we performed a one-way repeated-measures ANOVA on the microstate temporal results of the two experimental sessions (tPBM and sham) to test the period effects (Pre, Stim1, Stim2, Post). We verified the normality and homoscedasticity characteristics of the data to ensure that ANOVA usage was appropriate. Post-hoc pairwise comparisons were further carried out using Tukey’s adjustment for multiple variable comparisons to assess significant differences across 4 experimental periods (Pre, Stim1, Stim2, Post).

For the frequency-domain microstate analysis (Figure 2I), we employed the cluster-based permutation test (CBPT; Maris and Oostenveld, 2007; Oostenveld et al., 2011; Pellegrino et al., 2016; Benavides-Varela and Gervain, 2017) to compare the normalized frequency-specific power of four microstate classes between tPBM and sham sessions. This analysis enabled us to assess the significant differences in microstate power between tPBM and sham sessions across these four microstate classes.

## 3. Results

### 3.1. Alterations of EEG microstate topographies induced by tPBM

**Figure 3:**
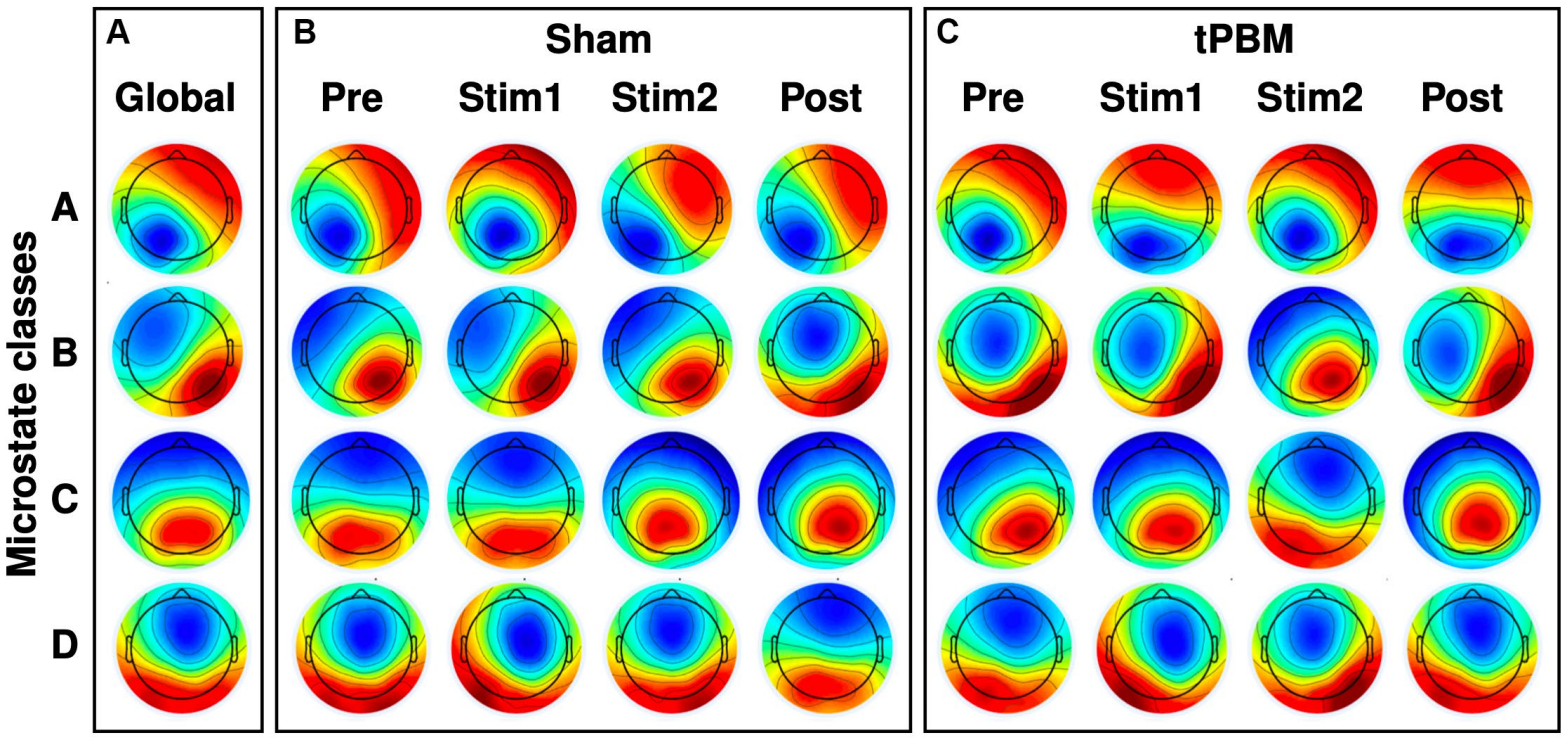
EEG microstate topographies of 4 microstate classes. **(A)** Global microstate topographies obtained from both tPBM and sham sessions during four experimental periods (Pre, Stim1, Stim2, and Post). **(B)** Microstate topographies during the four temporal segments under Sham session. **(C)** Microstate topographies during the four temporal segments under active tPBM session.

The four most dominant EEG microstate classes (A, B, C, and D) were identified under different conditions, and the respective microstate topographies are presented in Figure 3. Specifically, Figure 3A shows four microstate topographies derived from all the temporal segments and two tPBM/sham sessions. Figures 3B,C illustrate the time-dependent topographies under sham and tPBM interventions across all four microstate classes. These figures clearly display that microstate A exhibited a left occipital to right frontal polarity orientation, while microstate B presented a right occipital to left frontal orientation. Microstates C and D revealed roughly symmetric occipital to frontal and central to frontal polarity patterns, respectively.

### 3.2. Alterations of EEG microstate parameters induced by tPBM

As mentioned in Section 2, the labeled microstate time series were obtained by fitting the global microstate classes to each subject’s EEG data (Figure 2C). By using the microstate sequences, we computed several key temporal microstate parameters of the four experimental periods (Pre, Stim1, Stim2, and Post) for the tPBM and sham sessions. One-way repeated-measures ANOVA (rmANOVA) enabled us to reveal significant tPBM-induced changes in two key microstate temporal parameters throughout different experimental periods or segments for both tPBM and sham sessions.

**Figure 4:**
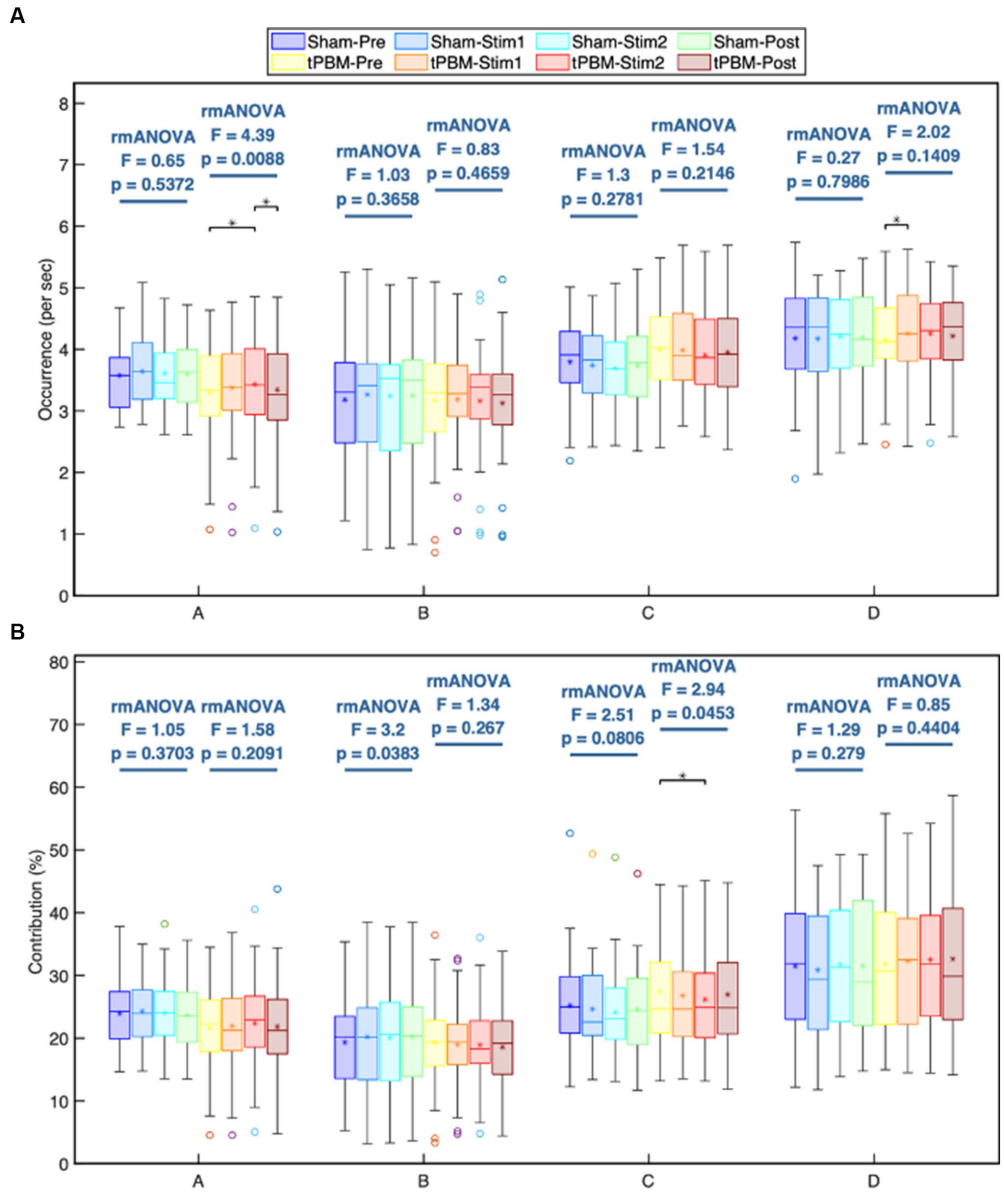
Statistical comparisons of the microstate **(A)** occurrence (1/s) and **(B)** contribution (%) parameters among four temporal segments (Pre, Stim1, Stim2, and Post) for each of the four microstate classes, A, B, C, and D. These comparisons are made independently for the active and sham sessions. Statistical results were obtained by one-way repeated measures ANOVA and the *post hoc* pairwise comparisons with Tukey correction. Significant differences between a period pair are marked as “∗” for *p* < 0.05 after Tukey correction.

Figures 4A,B depict the occurrence (per sec) and contribution (in %) of the four microstate classes throughout the different experimental periods (Pre, Stim1, Stim2, and Post) of the tPBM and sham sessions. The post-hoc rmANOVA tests along with Tukey’s method revealed a significant increase in the occurrence of microstates A and D and a significant decrease in the contribution of microstate C during the tPBM session. Specifically, the occurrence of microstate A gradually increased during the tPBM stimulation, leading to a significant difference between the Pre and Stim2 periods (*p_Tukey_* = 0.022). During the Post period, the occurrence of microstate A decreased significantly compared with that during Stim2 (*p_Tukey_* = 0.049). The occurrence of microstate D also increased notably during Stim1 of the tPBM session (*p_Tukey_* = 0.038 compared to Pre).

For the contribution parameter, the *post hoc* rmANOVA results showed that the contribution of microstate C of the tPBM session was significantly decreased during Stim2 compared to the baseline (Pre; *p_Tukey_* = 0.045). Regarding the sham condition, the rmANOVA analysis revealed significant differences in the contribution parameter of microstate B across four periods (p_rmANOVA_ = 0.038). However, the *post hoc* test did not identify any significant differences for six pairs of periods. The smallest *p_Tukey_* value for this case was 0.125 between the Pre and Stim1 periods. For other microstates, the statistical analysis did not reveal any significant differences across the four periods of both experimental sessions.

### 3.3. Alterations in transition probabilities among microstate classes induced by tPBM

**Figure 5:**
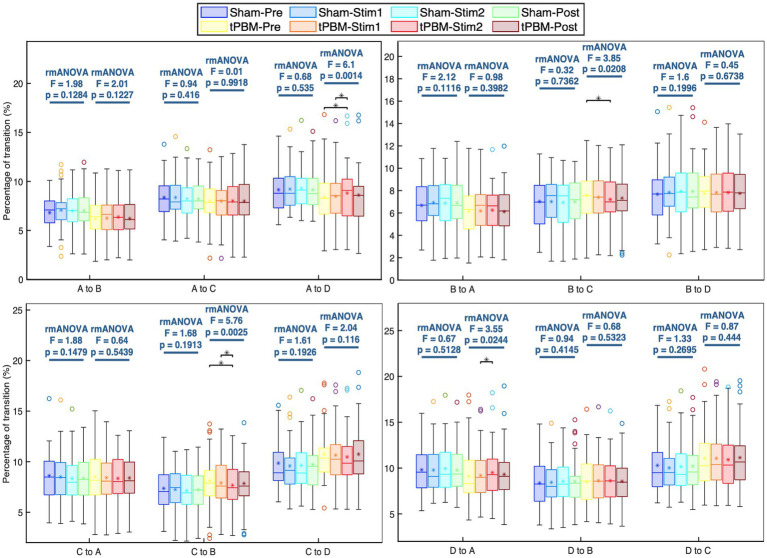
Transition probabilities between each pair of the four microstate classes and respective statistical comparisons under separate active and sham conditions. Statistical results were obtained by one-way repeated measures ANOVA and *post hoc* pairwise comparisons with Tukey’s correction. Significant differences between respective pairs are marked as “*” for *p* < 0.05, after Tukey’s correction.

Further analysis of the transition probabilities between different microstate classes revealed several significant differences induced by tPBM. Figure 5 shows the transition probabilities among the four microstate classes. Statistical results showed that the transition between microstates A and D increased significantly during the active stimulation period, whereas the transition between microstates B and C declined significantly. Specifically, the *post hoc* Tukey’s test revealed significant increases in the transition probabilities from microstate A to D between the Pre and Stim 2 periods (*p_Tukey_* = 0.01) and Stim1 and Stim2 periods (*p_Tukey_* = 0.013). The transition from microstate D to A also significantly increased between Stim1 and Stim2 (*p_Tukey_* = 0.041). In contrast, the transition probabilities from microstate B to C significantly decreased (*p_Tukey_* < 0.05, when comparing the Pre and Stim2 periods). Similarly, a significant drop in the transition probabilities from microstate C to B was also observed (*p_Tukey_* = 0.01 for Pre and Stim2 periods and *p_Tukey_* = 0.04 for Stim1 and Stim2 periods). For the sham session, the statistical analysis did not reveal any significant differences in the transition probabilities across the four temporal periods.

### 3.4. Influences of tPBM on EEG microstate topographical power

**Figure 6:**
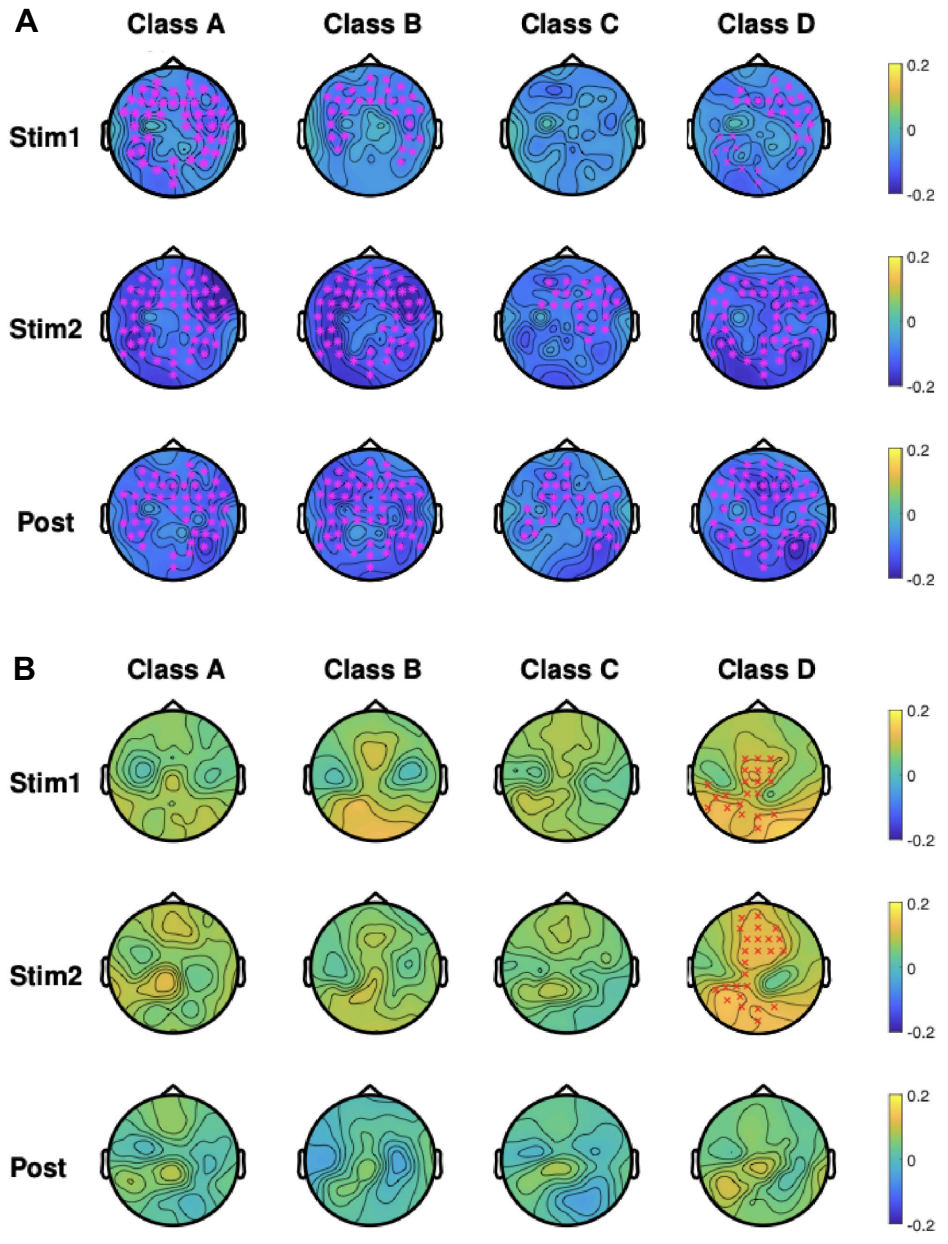
Topographic maps of group-averaged (*n* = 45), baseline-normalized changes in microstate power in four microstate classes during tPBM/sham and post-tPBM/sham periods in the **(A)** delta band and **(B)** alpha band. Stars/crosses indicate clusters of electrodes with significant differences between the conditions (“*” for *p*_cluster_ *<* 0.01 and “x” for *p*_cluster_ *<* 0.05). The purple color of the stars/crosses in **(A)** indicates that the normalized delta powers in the tPBM session were significantly lower than those in the sham session. The red color of the crosses in **(B)** indicates that the normalized alpha powers of the tPBM session were significantly higher than those in the sham session.

As mentioned in section 2, we performed EEG microstate analysis in the frequency domain and calculated the percentage changes in power for each microstate across the delta (0.5–4 Hz), theta (4–8 Hz), alpha (8–13 Hz), and beta (13–30 Hz) frequency bands using Eq. (4). Accordingly, Figures 6A,B show the baseline-normalized changes in microstate power across the delta band (Figure 6A) and alpha band (Figure 6B) for the four microstate classes during the tPBM and post-tPBM periods. The topographical maps also highlight channels whose cluster-associated *p*-values were below 0.05.

CBPT revealed significant differences in normalized power between the tPBM and sham sessions across multiple microstates. Specifically, within the delta band, the results revealed significantly lower normalized powers during and after tPBM compared to the sham session across all four microstate classes. In contrast, in the alpha band, the microstate powers of class D exhibited significant augmentation during the active tPBM session (Stim 1 and Stim 2) compared to the sham session. This augmentation, prominently observed in the central to the left-parietal region during Stim1 and in the mid-frontal to the left-parietal region during Stim2, underscores the distinctive impact of tPBM on microstate D in the alpha band.

## 4. Discussion

### 4.1. Effects of tPBM on EEG microstate classes and respective brain networks

Regarding EEG microstate classes, several simultaneous EEG-fMRI studies have investigated correlations between EEG microstates and fMRI resting states (Smith et al., 2009; Britz et al., 2010; Musso et al., 2010). Accordingly, microstate A is related to the activation of the bilateral superior and middle temporal lobes; microstate B is associated with the activation of the bilateral occipital cortex (Smith et al., 2009; Britz et al., 2010; Michel and Koenig, 2018). In addition, microstate C is linked to the dorsal anterior cingulate cortex, bilateral inferior frontal cortices, and right insular area, while microstate D is correlated with activation in the right-lateralized dorsal and ventral areas of the frontal and parietal cortices (Smith et al., 2009; Britz et al., 2010). Compared with prior publications in the literature, the four EEG microstate classes identified in this study are in good agreement with previous findings (Koenig et al., 2002; Michel and Koenig, 2018).

As shown in section 3, the statistical analysis of the temporal and spectral characteristics of these four microstates revealed that tPBM mainly modulated microstates A and D. This set of modulations implies that tPBM has the ability to alter or stimulate the resting human brain in the frontal, temporal, and parietal cortices. A recent human study (Dmochowski et al., 2020) that employed BOLD-fMRI to assess tPBM-induced hemodynamic activity found increases in resting-state functional connectivity in seed regions in the frontal, temporal, and parietal cortices. Another report from our own group developed a combined analysis of Singular Value Decomposition and eLORETA (Wang et al., 2022b), which revealed a tPBM-induced enhancement in alpha power in the frontal–parietal network. Thus, our findings derived from the EEG microstate analysis supplemented prior findings in brain regions stimulated by tPBM.

### 4.2. Effects of tPBM on EEG microstate classes and respective brain networks

As presented in sections 3.2 and 3.3, the results showed significant increases in (i) the occurrence of microstates A and D and (ii) the transition between microstates A and D during the stimulation period of the active tPBM session. These findings imply that tPBM promotes brain activity in microstates A and D, as well as more frequent transitions between them, all of which can also be considered potential indicators of active neuromodulation effects of tPBM. Previous studies on the functional significance of EEG microstates (Britz et al., 2010; Milz et al., 2016; Seitzman et al., 2017) have suggested that microstate A is associated with the auditory network, while microstate D is related to the dorsal attention network (DAN). A recent study using resting-state fMRI (Argilés et al., 2022) also reported an alteration in the functional connectivity of the DAN after red light exposure. Moreover, several papers have reported significant enhancement of attention and memory induced by tPBM (Barrett and Gonzalez-Lima, 2013; Hwang et al., 2016; Vargas et al., 2017; Jahan et al., 2019). In particular, Zhao et al. recently demonstrated that right-forehead tPBM with a 1,064-nm laser significantly enhances visual working memory capacity based on neuropsychological measurements of occipitoparietal contralateral delay activity (CDA; Zhao et al., 2022), which is well accepted as a robust neural correlate of visual working memory. Thus, we speculate that the tPBM-promoted increase in activity in microstates A and D may be a potential mechanism for brain function enhancement.

### 4.3. Alterations by tPBM in EEG microstate topographical delta and alpha powers

In addition to the time-domain EEG microstate analysis, we assessed the spectral information of microstate classes throughout the different experimental periods of both tPBM and sham sessions. To the best of our knowledge, only a few studies have focused on frequency-domain analysis of EEG microstates (Li et al., 2021). By combining the MEMD and HHT methods (Cho et al., 2017; Yang and Ren, 2019), we could overcome the tribulation due to the short-length signals of EEG microstates and estimate the frequency-specific power of different microstate classes. Accordingly, microstate spectral analysis revealed significant differences in the normalized power between the tPBM and sham sessions. Specifically, we observed that active tPBM induced significant reductions in normalized delta power in three microstates (A, B, and D). This aligns with prior findings (Jahan et al., 2019; Wang et al., 2021; Shahdadian et al., 2022) that have also reported a decrease in delta power during and after tPBM. For instance, a previous study demonstrated a significant decline in delta power in the tPBM group, as opposed to a notable increase in delta power in the sham group (Jahan et al., 2019). These observations resonate with previous EEG studies that have inferred a connection between the increase of slow wave oscillations and an individual’s proclivity for rest and sleep. Thus, tPBM emerges as a potential mitigator of neuronal fatigue through its capacity to augment cellular energy production and promote neuronal metabolism.

Microstate spectral analysis also unveiled that tPBM significantly enhanced normalized alpha power topographies in microstate D during the active tPBM period. In particular, enhanced powers occurred in the central to left-parietal region during Stim1 and in the mid-frontal to left-parietal region during Stim2. This latter finding underscores the importance of both alpha power and microstate D. Because microstate D is closely associated with brain activity in the dorsal and ventral areas of the frontal and parietal cortices (Smith et al., 2009; Britz et al., 2010), our results imply that right-prefrontal tPBM facilitates significant promotion of EEG activity in microstate D across the frontal and parietal regions in alpha rhythm. Overall, both time-domain and frequency-domain EEG microstate analyses presented us with the same key microstate class, namely, class D, which was most significantly modulated by the right-forehead tPBM with a 1,064-nm laser compared to other EEG microstate classes.

It is worth noting that although the local stimulation site experienced a slight increase in skin temperature due to light absorption, the changes in microstate parameters observed in this study were not a result of the thermal effect caused by light. Studies on the effects of tPBM on brain temperature, conducted through a computational model (Bhattacharya and Dutta, 2019) and magnetic resonance thermometry (Dmochowski et al., 2020), revealed no significant difference in temperature between tPBM and sham conditions. Additionally, a recent EEG study comparing tPBM and thermal stimulation found notable differences in the alterations of EEG power topography between the two types of stimulation (Wang et al., 2021).

### 4.4. Limitations and future work

While this study has enabled us to obtain several new findings, several limitations exist. First, in Session 4.2, we attempted to elucidate the impact of tPBM on microstate parameters by considering the roles of microstates suggested by previous studies in the literature. However, the specific function of microstates may vary depending on the circumstances in which the EEG data was collected. Second, we took the ICA-based artifact correction approach, which could create an author-made artifact and thus affect the validity of the study. Third, we chose to adopt four microstate classes, as mostly employed in prior EEG microstate studies. Nevertheless, we acknowledge that integrating formal criteria to determine the optimal number of microstates holds the potential to bolster the robustness of the analysis. Last, it is possible that tPBM may affect the occurrence of artifacts since some of them are related to brain behaviors.

To overcome the limitations, further work includes (1) to conducting source localization analysis that can provide a more comprehensive explanation of the changes in microstate parameters throughout the tPBM session and further insights into its effects; (2) to perform artifact rejections to minimize potential confounds introduced by artifact correction methods; (3) to introduce a more systematic procedure for microstate selection to characterize more comprehensively/accurately the microstate patterns present in the EEG data; and (4) to investigate tPBM-induced artifacts on EEG microstates.

## 5. Conclusion

In this study, 64-channel EEG data were recorded from 45 healthy subjects under both active and sham 8-min tPBM with a 1,064-nm laser delivered on the right forehead of the subjects. Both time- and frequency-domain analyses were employed to identify and investigate tPBM-induced alterations in the dynamic EEG microstates in the human brain. Four global microstate classes for both the tPBM and sham sessions throughout the different experimental periods (i.e., pre-, during, and post-stimulation) were first identified using conventional EEG microstate analysis. Various temporal microstate parameters were then extracted and statistically analyzed to assess the effects of tPBM on temporal brain dynamics. Moreover, spectral analysis was also performed to investigate the variation in EEG power of microstate classes over the respective periods of tPBM and sham sessions. Statistical analyses revealed that tPBM resulted in (1) a significant increase in the occurrence of microstates A and D and a significant decrease in the contribution of microstate C; (2) a substantial increase in the transition probabilities between microstates A and D; and (3) a substantial increase in the alpha power of microstate D. These findings not only were consistent with our previous reports on tPBM-induced alterations in EEG power, but also confirmed the neurophysiological effects of tPBM on EEG microstates, particularly in class D, which reflects brain activation across the frontal and parietal regions. Future efforts should include investigations of the relationships between cognition-evoked functional improvement and alterations of EEG microstates in response to tPBM for a better understanding of the underlying mechanism between them.

## Data availability statement

The raw data supporting the conclusions of this article will be made available by the authors, without undue reservation.

## Ethics statement

The studies involving humans were approved by the Institutional Review Board of the University of Texas at Arlington, Arlington, TX, USA. The studies were conducted in accordance with the local legislation and institutional requirements. The participants provided their written informed consent to participate in this study.

## Author contributions

NT analyzed the data, interpreted the results, and prepared the manuscript. XW assisted with data collection, discussed the results, and reviewed the manuscript. HL initiated and supervised the study, discussed and interpreted the results, as well as reviewed and revised the manuscript. All authors contributed to the article and approved the submitted version.
