# Supplementary material for: Temporal and spectral analyses of EEG microstate reveals neural effects of transcranial photobiomodulation on the resting brain
Source: Front Neurosci. 2023 Oct 17;17:1247290. doi: 10.3389/fnins.2023.1247290 (PMC10616257; doi:10.3389/fnins.2023.1247290)
Supplement: Supplementary file 1 [file Data_Sheet_1.docx]

Temporal and Spectral Analyses of EEG Microstate Reveals Neural Effects of Transcranial Photobiomodulation on the Resting Brain

Nghi Cong Dung Truong, Xinlong Wang, and Hanli Liu

***Steps used to perform EEG microstate analysis in the frequency domain***

Following the methodology described by (Li et al., 2021), we performed frequency-domain EEG microstate analysis as follows.

Step 1: We employ a multivariate empirical mode decomposition (MEMD) algorithm to decompose the N-channel EEG signal into a set of intrinsic mode functions (IMFs) representing different oscillatory frequencies or levels embedded in the original signal. MEMD is an extended method of EMD, the latter of which is a data decomposition method for non-linear and non-stationary signals (Huang et al., 1998). EMD enables any complicated dataset to be expressed using a finite number of IMFs. The MEMD was developed by taking signal projections along different directions in *N*-dimensional spaces, a generalization of EMD (Rehman and Mandic, 2010).

According to ref. (Li et al., 2021, Rehman and Mandic, 2010), multiple N-dimensional envelopes were generated by taking N-channel EEG signal projections along respective directions in the N-dimensional space and then averaged to obtain the local mean. Let $s\left( t \right)=\left[ s_{1}(t),s_{2}(t),\ldots,s_{\left\{ N \right\}}(t) \right]$ be N-channel EEG signals and $\{v^{\theta^{k}}= \left[ v_{1}^{k}, v_{2}^{k},\ldots,v_{\left\{ N \right\}}^{k} \right]{\}}_{k=1}^{K}$ denote a set of direction vectors along the direction given by angles $\left\{ \theta^{k}= \left[ \theta_{1}^{k},\theta_{2}^{k},\ldots,\theta_{\left\{ \left( N-1 \right) \right\}}^{k} \right] \right\}_{k=1}^{K}$ on a (N-1) sphere, where K represents points of sampling on the sphere uniformly. The EEG signal s(*t*) was projected along each direction vector $v^{\theta^{k}}$, and the maxima $t_{i}^{\theta^{k}}$ of the projected signals were located. The multivariate envelopes $e^{\theta^{k}}\left( t \right)$ were estimated by interpolating $[t_{i}^{\theta^{k}}, s\left( t_{i}^{\theta^{k}} \right)$] via cubic splines. The local mean of the multidimensional envelopes was finally computed as:

$m\left( t \right)=\frac{1}{K}\sum_{k=1}^{K} e^{\theta_{k}}\left( t \right).$ (1)

After the mean signal was defined, the “detail signal” *d*(*t*) was extracted using *d*(*t*)=*s*(*t*) *− m*(*t*). If *d*(*t*) fulfills the stopping criterion for a multivariate IMF (Rehman and Mandic, 2010), *d*(*t*) is chosen as the IMF. Otherwise, let *s*(*t*) = *d*(*t*), and the procedure is repeated until the generated signals become monotonic or do not contain sufficient extrema to form a meaningful multivariate envelope. This step is illustrated in Fig. 2(f).

Step 2: The Hilbert transform is then applied to each IMF to obtain the instantaneous phase and frequency (Li et al., 2021, Rehman and Mandic, 2010). The Hilbert transform on *n^th^* channel for *i^th^* IMF $d_{i}^{n}\left( t \right)$ is given by

$H\left( d_{i}^{n}\left( t \right) \right)=\frac{1}{\pi}P\int_{-\infty}^{\infty} \frac{d_{i}^{n}\left( \tau\right)}{t-\tau}d\tau$, (2)

where *P* is Cauchy’s principal value. Accordingly, the amplitude $a_{i}^{n}\left( t \right)$, phase $\theta_{i}^{n}\left( t \right)$, and frequency functions $\omega_{i}^{n}\left( t \right)$will be quantified using the following equations for each microstate spectral analysis:

$a_{i}^{n}\left( t \right)=\left| d_{i}^{n}\left( t \right)+jH\left( d_{i}^{n}\left( t \right) \right) \right|,$ (3)

$\theta_{i}^{n}\left( t \right)=\arctan\left( \frac{H\left( d_{i}^{n}\left( t \right) \right)}{d_{i}^{n}\left( t \right)} \right)$, (4)

$\omega_{i}^{n}\left( t \right)=\frac{d\theta_{i}^{n}\left( t \right)}{dt}$. (5)

Accordingly, the Hilbert spectrum *H^n^*(ω, *t*) for the *n^th^* EEG channel is computed as:

$H^{n}\left( \omega,t \right)=Re\left\{ \sum_{i=1}^{M} a_{i}^{n}\exp\left( j\int\omega_{i}^{n}\left( t \right)dt \right) \right\}$. (6)

This step is depicted in Fig. 2(g); it shows a time-frequency map for channels 1 and n.

Step 3: The power for the microstate *m* of the *n^th^* channel in the frequency band (*<fb>*) is calculated as follows:

$P_{<fb>}^{mn}=\frac{1}{\Delta\omega}\frac{1}{L_{m}}\iint_{\Delta\omega L_{m}} H^{n}(\omega,t)^{2}dtd\omega$ (7)

where *L_m_* is the total temporal length of the microstate *m* and ∆ω is the range of the frequency band <fb>. Fig. 2(h) shows schematically power topographies of microstate classes A, B, C, and D in delta (δ: 0.5-4 Hz), theta (θ: 4-8 Hz), alpha (α: 8-13 Hz), and beta band (β: 13-30 Hz).

**References:**

HUANG, N. E., SHEN, Z., LONG, S. R., WU, M. C., SHIH, H. H., ZHENG, Q., YEN, N.-C., TUNG, C. C. & LIU, H. H. 1998. The Empirical Mode Decomposition and the Hilbert Spectrum for Nonlinear and Non-Stationary Time Series Analysis. *Proceedings of the Royal Society of London. Series A: Mathematical, Physical and Engineering Sciences,* 454**,** 903–995-903–995.

LI, Y., SHI, W., LIU, Z., LI, J., WANG, Q., YAN, X., CAO, Z. & WANG, G. 2021. Effective Brain State Estimation During Propofol-Induced Sedation Using Advanced EEG Microstate Spectral Analysis. *IEEE Journal of Biomedical and Health Informatics,* 25**,** 978–987-978–987.

REHMAN, N. & MANDIC, D. P. 2010. Multivariate Empirical Mode Decomposition. *Proceedings of the Royal Society A: Mathematical, Physical and Engineering Sciences,* 466**,** 1291–1302-1291–1302.
